# Nevus Senescence

**DOI:** 10.5402/2011/642157

**Published:** 2011-06-22

**Authors:** Andrew L. Ross, Margaret I. Sanchez, James M. Grichnik

**Affiliations:** ^1^Department of Dermatology and Cutaneous Surgery, University of Miami Miller School of Medicine, Miami, FL 33136, USA; ^2^Melanoma Program, Sylvester Comprehensive Cancer Center, Miller School of Medicine, University of Miami, Miami, FL 33136, USA; ^3^Interdisciplinary Stem Cell Institute, Miller School of Medicine, University of Miami, Miami, FL 33136, USA

## Abstract

Melanomas and nevi share many of the same growth-promoting mutations. However, melanomas grow relentlessly while benign nevi eventually undergo growth arrest and stabilize. The difference in their long-term growth potential may be attributed to activation of cellular senescence pathways. The primary mediator of senescence in nevi appears to be p16. Redundant, secondary senescence systems are also present and include the p14-p53-p21 pathway, the IGFBP7 pathway, the FBXO31 pathway, and the PI3K mediated stress induced endoplasmic reticulum unfolded protein response. It is evident that these senescence pathways result in an irreversible arrest in most instances; however, they can clearly be overcome in melanoma. Circumvention of these pathways is most frequently associated with gene deletion or transcriptional repression. Reactivation of senescence mechanisms could serve to inhibit melanoma tumor progression.

## 1. Introduction

Melanocytic neoplasms represent a diverse group of tumors that can be either benign (nevi) or malignant (melanoma). The most striking difference between benign and malignant melanocytic neoplasms is that the former eventually stabilize and undergo cellular senescence while the latter continue to grow. It is evident that cellular senescence, loosely defined as an arrested proliferative capacity, is governed by multiple mechanisms. It is becoming clear that these mechanisms represent the cellular processes that differentiate nevi from melanomas. In this paper, the conceptual framework for nevus growth will be reviewed along with what is known about cellular senescence pathways that terminate nevus growth.

## 2. Nevus Life Cycle

It is hypothesized that nevi originate secondary to a mutation sustained in a single progenitor cell [1]. This mutation then induces the progenitor cell to develop into a nevus that follows an archetypal life cycle. The stages of this cellular life cycle can be separated into the phases of initiation, promotion, growth termination, and involution [1]. 

Initiation occurs when the nevus progenitor cell acquires a mutation. The mutated cell then remains quiescent and inconspicuous. Promotion occurs when the mutated cell is stimulated to undergo proliferation. This unmasks the mutation, which in turn causes melanocytic nevus cells to accumulate. It is not known exactly what drives this process. However, it is significant that the majority of nevi develop in late childhood and young adulthood. Presumably, the endogenous factors that promote maturation of the child into an adult also promote the growth of nevi. Some insight into this process may be gleaned from the study of eruptive nevi wherein immunomodulatory agents and cytokines are thought to promote their growth. 

Growth termination begins to occur as the nevi mature. A number of molecular pathways are involved in growth termination and current knowledge will be reviewed in more detail below. Involution occurs when the growth-arrested nevus begins to regress and eventually disappears. This may occur through a number of processes including apoptosis, immune destruction, or withdrawal of growth factors like MSH [2–4]. It is interesting to note that while late childhood and young adulthood represents the time period in which the majority of nevus promotion occurs, it is also the time period in which most nevi begin to involute [5]. Thus, it is possible that nevi still present in late adulthood represent nevi that possess cellular mechanisms that make them resistant to involution. It remains important to understand these mechanisms in order to develop strategies to block growth, induce senescence, and promote involution.

## 3. Defining Senescence

Senescence is said to occur when a cell exits the cell cycle and stops proliferating. In melanocytes, this growth arrest is accompanied by a number of morphological and functional changes. These changes include adoption of a large, flat, sometimes vacuolated appearance, alterations in chromatin structure, differential gene expression patterns, and production of senescence-associated-beta-galactosidase (SA-*β*-Gal) [6–10]. Of note, alternations in growth media conditions, molecular mutations, and knock out models have been shown to allow cells to either partially or completely overcome senescence. 

The basic question remains: what is senescence and is it really permanent? Nonproliferating cells capable of readily exiting and reentering the cell cycle are often referred to as quiescent. Consequently, it is necessary to differentiate senescence from quiescence. Many would argue that the major difference between quiescent and senescent cells is that the former will proliferate in response to a mitogenic signal while the latter will not [11, 12]. This occurs because the phenomenon of quiescence is associated with reversible epigenetic silencing of transcription through reversible histone modification [13].

Furthermore, it has been argued that the discovery of “irreversible” transcriptional silencing through the development of senescence associated heterochromatin foci (SAHF) [14] distinguishes a senescent cell from a quiescent cell. While it is true that SAHF is a salient feature of senescent cells and that it more readily prohibits transcription than the histone modifications present in quiescent cells, the irreversibility of SAHF has been questioned [15]. This suggests that there are either more complex mechanisms permanently keeping cells from proliferating or that senescence is reversible, just to an exponentially lesser extent than quiescence.

## 4. Telomeres and Nevus Senescence

It is well accepted that telomeres in somatic cells undergo shortening with each successive cellular division. This progressive reduction in size culminates in growth arrest. It has also been shown that the enzyme telomerase functions to extend telomeres, thus permitting continuous cellular divisions [17]. Subsequently, it was shown that telomerase activity is increased in germ cells and immortal cancer cell lines [18]. This occurs because these cells express telomerase reverse transcriptase (hTERT), the catalytic subunit of telomerase that is absent in somatic cells.

Telomere shortening is known to promote melanocyte senescence [19]. It has also been shown that melanocytes supplemented with hTERT can escape normal senescence [19]. Therefore, it is not surprising that benign nevi demonstrate no telomerase activity while over 90% of melanomas do [20]. This suggests that once melanocytes within nevi undergo a certain number of divisions, their telomeres will reach a critical size that induces growth arrest and senescence.

It is also interesting to note that there is a strong correlation between nevus count, nevus size, and systemic telomere length [21]. This suggests that melanocytic cells in individuals with long telomeres are able to undergo a greater number of cell divisions before their telomeres shrink to the critical size that induces senescence. This in turn not only allows more nevi to develop but also permits them to reach larger diameters before undergoing senescence [21]. Consequently, telomere shortening appears to represent one mechanism that signals growing melanocytes within nevi to exit the cell cycle and undergo senescence.

## 5. Molecular Mediators of Telomere-Dependent Cellular Senescence

Most of our knowledge pertaining to molecular senescence initially was derived from the study of mouse and human fibroblasts. Although human melanocytic senescence pathways deviate from these more traditional models, a brief review of their function is useful in understanding melanocyte senescence. Fibroblasts were initially thought to possess two distinct phases of senescence known as mortality phase 1 (M1) and mortality phase 2 (M2). Each of these phases is regulated by molecular pathways and cellular events that prevent cells from undergoing cell cycle progression. 

**Figure 1:**
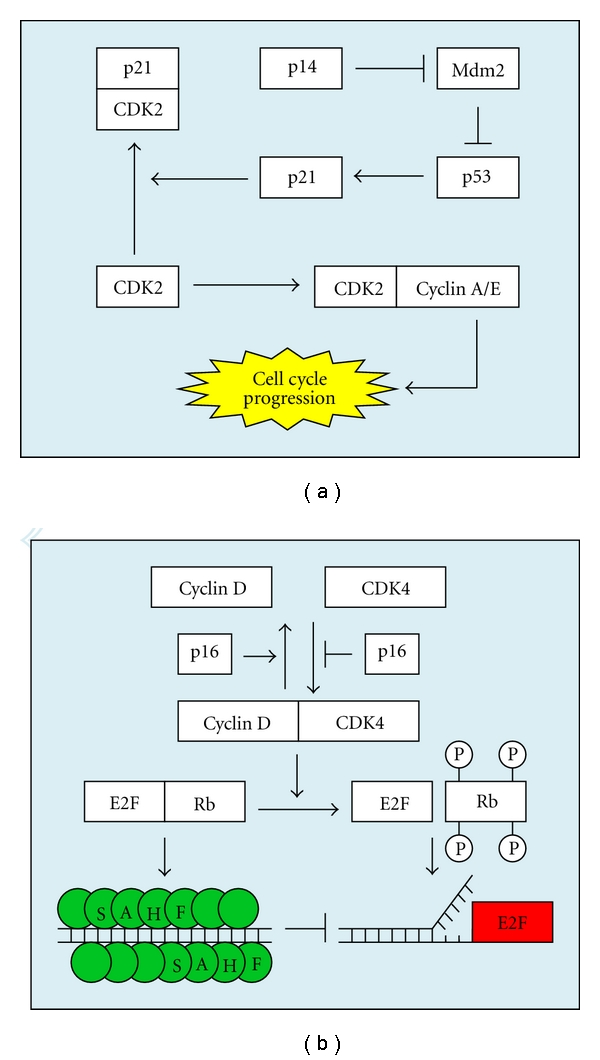
The p14-p53-p21 pathway and the p16-Rb signaling pathways involved in fibroblast senescence. (a) Activated p53 induces expression of p21. The protein p21 binds to cyclin-dependent kinase 2 (CDK2), which in turn impedes CDK2 from complexing with Cyclin E and Cyclin A. Since the CDK2-Cyclin E/A complexes are required for DNA replication to begin, p21 effectively stops cell replication. Mdm2 is a strong antagonist of p53. It not only prevents production of new p53 through transcriptional inhibition, but also exports active p53 from the nucleus and targets it for proteolytic destruction through ubiquitination. The tumor suppressor protein p14 works to upregulate p53 by inhibiting Mdmd2 [16]. (b) While in its unphosphorylated state, Rb sequesters the transcription factor (E2F) responsible for initiating DNA replication. Unphosphorylated Rb also induces the formation of SAHF, which prevents free E2F from complexing with DNA. When cyclin-dependent kinase 4 (CDK4) complexes with cyclin D, it is able to phosphorylate Rb, thus releasing E2F and allowing DNA replication to occur. The protein p16 works as a tumor suppressor by preventing CDK4 from complexing with cyclin D.

This first phase, M1, is mediated by two major molecular pathways: the p53-p21 pathway (Figure 1(a)) and the p16-Rb pathway (Figure 1(b)). It was previously proposed that telomere shortening is responsible for induction of both of these pathways [22]. While this proved to be true, there exists some variation in pathway activation. It is well accepted that telomere shortening results in an upregulation of both p53 and p21 [23, 24]. There also exists ample evidence that shows p16 is upregulated in response to telomere shortening [25–27]. However, telomere-induced p16 expression occurs with delayed kinetics. Thus, it is apparent that telomere shortening is not the primary mechanism responsible for p16-Rb-mediated senescence. Consequently, p16-Rb has occasionally been referred to as the mediator of the telomere-independent pathway of senescence, despite the fact that it can be induced by telomere shortening [28]. Nevertheless, only one of these pathways is necessary to keep fibroblasts senescent in M1 [29]. Consequently, both pathways must be knocked out to allow cells to escape M1 senescence.

In the event that both of these pathways are inactivated, fibroblasts are able to overcome the first phase of senescence and continue to replicate for a finite number of divisions. At this point, the cells enter the second phase of senescence, M2. This is often referred to as “crisis.” This second phase of senescence occurs because the telomeres have become so short that they are no longer able to prevent end to end fusions of chromosomes. These fusions result in dicentromeric chromosomes that undergo an increased incidence of double stranded DNA breaks. In turn, these breaks prevent further cellular divisions.

## 6. Mitogen- and Oncogene-Induced Senescence

It has become readily apparent that there exists another phase of senescence that occurs before M1. This phase of senescence, first referred to as M0, is thought to be largely dependent on the p16-Rb pathway [30] and occurs independently of telomere shortening [31]. Other molecular mediators, such as p14 and p53, have also been implicated in M0 [32]. Recently, it has been shown that neither pharmacologic inhibition of DNA damage nor direct antagonism of p53 affects M0 senescence in nevi [33]. Furthermore, disruption of the p16 pathway in M0 arrested keratinocytes resulted in the recommencement of cellular division for a finite number of cycles that eventually underwent p53 mediated M1 senescence. Consequently, it appears that the p16-Rb pathway is the primary mediator of M0 senescence. This phase of senescence is now known to be induced by over stimulation of mitogenic pathways [34]. This senescence phenomenon is now referred to as a telomere-independent mitogenic clock that can be abrogated by certain growth conditions [35] or induced by oncogenic signaling [36].

## 7. Molecular Melanocyte Senescence

Nevi often possess oncogenic mutations in proteins that participate in mitogenic signaling [1]. Consequently, it is not surprising to find that nevi undergo M0 mitogenic senescence. Michaloglou et al. confirmed this *in vivo *by demonstrating that BRAF V600E mutant-positive nevi have increased p16 and SA-*β*-Gal expression [37]. Furthermore, these nevi had an increased number of SAHF and did not possess critically shortened telomeres. Taken together, this evidence confirms that nevi undergo a telomere-independent p16-mediated mitogenic senescence when the BRAF V600E mutation is present. 

However, Michaloglou et al. also demonstrated that islands of senescent melanocytes within the BRAF V600E nevi did not have high expression patterns of p16. One explanation for heterogeneous expression of p16 in senescent nevi involves the observation that not all melanocytes within V600E mutant-positive nevi possess the mutation [38]. Consequently, the islands of cells not expressing p16 may not have contained the V600E BRAF mutation. However, another study demonstrated that p16 induction is not required for BRAF V600E-mediated senescence to occur [39]. It is thus most likely that other molecular mediators are also involved in BRAF V600E-induced senescence. 

Other studies have shown that BRAF V600E mutations induce senescence through upregulation of insulin-like growth factor-binding protein 7 (IGFBP7) [40]. IGFBP7 works to inhibit mitogenic RAF-MEK-ERK signaling through autocrine and paracrine stimulation. Consequently, it is not surprising to find that only 23% of BRAF V600E-positive nevi have detectable levels of ERK, while 93% of BRAF V600E-positive melanomas have detectable levels of ERK [41]. It is thus possible that the IGFBP7 pathway is lost in melanoma and that this could contribute to overcoming senescence. 

The cellular response to DNA damage induced by hyperreplication of cells exposed to mitogenic overstimulation also plays a role in mediating senescence [42, 43]. This has been shown to occur through destruction of Cyclin D1 by the FBXO31 protein in BRAF V600E-positive melanocytes [44]. So while the p16 pathway may play a significant role in BRAF-induced senescence, it is clear that other mechanisms exist. Consequently, p16 may not be necessary for BRAF-induced senescence.

HRAS-induced senescence exhibits a number of markedly distinct characteristics when compared to BRAF-induced senescence. For example, HRAS mutations induce senescence more rapidly than BRAF mutations in human melanocytes [45]. Additionally, it has been demonstrated that melanocytes that undergo HRAS-induced senescence display specific microscopic features, like extensive vacuolization, a finding not known to occur in BRAF-induced senescence. Further investigation of this phenomenon demonstrated that these features are secondary to a PI3K pathway mediated stress induced endoplasmic reticulum unfolded protein response [45]. Thus, it is not surprising that there is a phenotypic variation in HRAS and BRAF senescence phenotypes given the fact that HRAS is upstream of PI3K, while BRAF is not. At first glance, it is a little more surprising that NRAS-induced senescence (NRAS is an isoform of HRAS that is able to activate identical downstream effectors) does not display the HRAS-induced senescence phenotype. However, this finding is readily explained by the fact that HRAS has a much greater affinity for PI3K [46]. 

Despite the fact that HRAS can induce a phenotypically distinct form of senescence, it appears that HRAS-positive nevi are still governed by more traditional modes of senescence. For example, it has been shown that the loss of the p16 locus prevents telomere-induced senescence in melanocytes with HRAS mutations [47]. Similar findings have been demonstrated in human fibroblasts with HRAS mutations [28]. The occurrence of p16-induced senescence and the unfolded protein response in HRAS mutant nevi supports the notion that there are multiple pathways and mediators that serve as repetitive safeguards against unchecked proliferation.

It is not unreasonable to put forth the notion that the NRAS-induced senescence phenotype may be considered a hybrid of BRAF and HRAS-induced senescence phenotypes. This is because while NRAS-induced senescence occurs with similar timing as BRAF-induced senescence, it displays a mild form of vacuolization [45]. These findings are likely secondary to RAS isoform specificity, with NRAS generating greater amounts of phosphorylated ERK and HRAS preferentially activating the PI3K pathway [45, 46].

There are a number of other important lessons that have been learned from melanocytes harboring NRAS mutations. For example, it has been shown that NRAS-induced senescence is associated with DNA damage that is postulated to upregulate both p16-Rb and p14-p53-p21 [33]. Haferkamp et al. also confirmed that while the p16-Rb pathway appears to be the more prominent mediator of senescence in melanocytes, the p53 pathway is capable of initiating a phenotypically identical, though delayed, form of senescence [33]. One of the most interesting findings was that while p16 is by no means required to induce senescence in NRAS mutant melanocytes [48], it was necessary to form SAHF [33]. Consequently, the possibility exists that melanocytes with defective p16-Rb cellular machinery may undergo incomplete, pathological forms of senescence that are more prone to melanomagenesis.

It has also been shown the potency of mitogenic pathway stimulation has an influential role in determining if a cell will senesce. Leikam et al. demonstrated that strong oncogenic signaling led to the development of a senescent multinucleated melanocyte population *in vitro* while weak signaling promoted proliferation [49]. Reactive oxygen species, previously known to be induced by the RAS-RAF-MEK-ERK pathway [50], were shown to be responsible for this senescent phenotype independent of p53 and Rb transcription levels. The authors also noted that the presence of this multinucleated phenotype precludes this form of senescence from occurring through G0 exit from the cell cycle commonly seen in M1. This is because the presence of multiple nuclei suggests that the cell has already passed the DNA synthesis checkpoint as it is actively replicating its DNA. This may explain the lack of correlation between this senescent phenotype and p53 and Rb expression. Consequently, the presence of multinucleated melanocytes in nevi may be evidence of yet another, non-G0 senescence mechanism meant to protect cells against overactive mitogenic signaling.

## 8. Breaking Senescence

**Figure 2:**
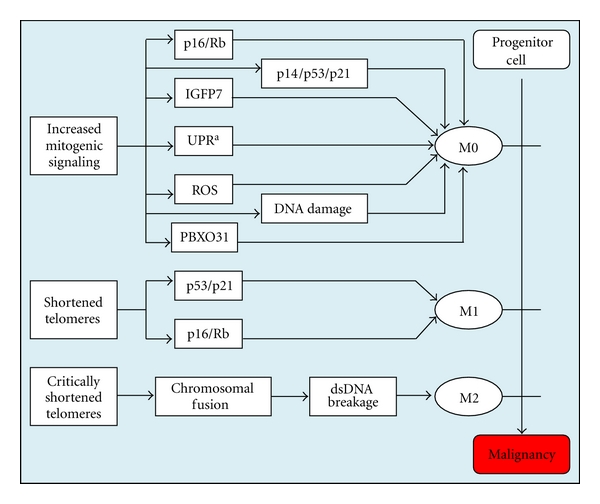
Overview of senescence pathways. Stimuli and cellular mechanisms responsible for the various stages of senescence. Each phase of senescence prevents cells from undergoing further cell division. M0 can occur through multiple mechanisms, and the relative contribution of each is a function of the factors driving mitogenic stimulation (e.g., BRAF versus HRAS mutation). M0 appears to be driven predominantly by the p16/Rb pathway in melanoma. a: Unfolded protein response (unique to HRAS mutant-positive cells).

It is evident that there exist multiple mechanisms through which nevi initiate senescence (Figure 2). This includes telomere shortening, mitogenic overstimulation, increased free radical production, and DNA damage. These stimuli trigger senescence through multiple, often shared molecular mechanisms that include induction of the p16-Rb pathway, the p14-p53-p21 pathway, the IGFBP7 pathway, the FBXO31 pathway, and the endoplasmic reticular unfolded protein response. The redundancy of these mechanisms likely evolved as a safeguard against tumor initiation. Given this redundancy, it is a little surprising that disruption of a single component of these pathways can both promote nevus formation and confer such a markedly increased risk of developing melanoma. 

Despite the fact that p16 and p14 share no protein sequence similarity, they are both encoded by a single gene locus known as cyclin-dependent kinase inhibitor 2A (CDKN2A) [51]. Mutations in this gene locus have been reported that can affect p16, p14, or both p16 and p14 [52]. The subsequent discussion refers to mutations that only affect one of these two proteins.

Individuals with systemic deactivating p16 mutations have been shown to possess a greater number of nevi [53]. They also continue to develop new nevi at a much faster rate than wild type p16 familial controls [54]. These findings are supported by a report of a pedigree harboring a heat sensitive deactivating mutation in p16 that developed a significantly larger number of nevi in sun exposed areas [55]. It is likely that melanocytes in these individuals fail to undergo M0 and continue to divide until their telomeres shorten to a point that M1 is initiated. Given the above, it is clear that disruption of the p16 pathway is sufficient to hinder or even prevent senescence in melanocytes. 

Nevi have been shown to express significantly more p16 than melanomas [56]. Straume et al. reported that 45% of primary melanomas and 77% of metastatic melanomas lacked expression of p16 [57]. Although this demonstrates that compromised p16 function is a predisposing factor for developing melanoma, it is not alone sufficient to initiate melanomagenesis. Instead, it appears that loss of p16 may be the transforming event that allows a benign nevus to transform into a melanoma, thus overcoming senescence. In such a model, loss of p16 expression could result in a slow loss of SAHF, thus allowing strong mitogenic signaling to eventually reactivate the cell cycle. While loss of p16 may be sufficient to allow continued nevus growth, multiple mutations must occur within the senescence pathways to induce melanomagenesis. 

Mutations in other molecular mediators of senescence like p14, p21, and p53 have been characterized in malignant melanocytic neoplasms. Isolated p14 mutations occur less frequently than p16 mutations [58, 59]. Like their p16 counterparts, individuals with a germline mutation affecting p14 are more susceptible to developing melanoma [60]. Though uncommon, it has been shown that p21 mutations can also be present in melanoma [61]. Interestingly, p21 is expressed with much greater frequency in melanomas (61%) than in nevi (28%) [62]. This suggests that p21 acts more as a failsafe mechanism designed to arrest cells that have transformed and escaped p16-mediated senescence than as a primary mediator of nevi senescence. Lastly, while p53 mutations have been described in melanoma, they occur infrequently [63] and are thought to play only a minor role in melanomagenesis [64]. However, recent evidence suggests that p53 may play an active role in preventing nevus progression to melanoma in the murine model [65]. Since p19 (the murine analogue of p14) and p53 have an inherently stronger role in inducing senescence in mice than in humans, the importance of this pathway in human melanoma remains unclear [66].

## 9. Conclusion

Senescence represents a dynamic, ongoing process with multiple stages and checkpoints that prevents cells from entering the cell cycle. As such, senescence acts as a barrier to uncontrolled tumor enlargement and malignant degeneration. Senescence pathways are activated in benign nevi where they work to prevent further growth. The p16 pathway appears to be the primary mediator of senescence in nevi. It seems that redundant, possibly secondary senescence systems are also present in nevi. These include the p14-p53-p21 pathway, the IGFBP7 pathway, the FBXO31 pathway, and the PI3K mediated stress induced endoplasmic reticulum unfolded protein response. It is evident that though senescence results in an irreversible arrest in most instances, it can clearly be overcome in pathological processes, like melanoma. While it is clear that these pathways are overcome with tumor progression, the stage of melanomagenesis during which the loss occurs is not known. However, the fact that most melanomas appear to arise de *novo* suggests that some of these pathways are overcome at very early stage. Continued research in this area will also help us to better differentiate benign and malignant tumors. It may also allow for the development of senescence inducing therapies to hinder the growth of melanoma cells.

##  Disclosures

 Digital Derm, Inc: major shareholder, spectral Image; Inc: past grants and consulting Genentech: J. M. Grichnik is a consultant, Archives of Dermatology, skin sight section editor.
